# Anthropogenic platinum group element (Pt, Pd, Rh) concentrations in PM_10_ and PM_2.5_ from Kolkata, India

**DOI:** 10.1186/s40064-016-2854-5

**Published:** 2016-08-02

**Authors:** Huey Ting Diong, Reshmi Das, Bahareh Khezri, Bijayen Srivastava, Xianfeng Wang, Pradip K. Sikdar, Richard D. Webster

**Affiliations:** 1 Earth Observatory of Singapore, Nanyang Technological University, Singapore, 639798 Singapore; 2 Asian School of the Environment, Nanyang Technological University, Singapore, 639798 Singapore; 3 Division of Chemistry and Biological Chemistry, School of Physical and Mathematical Sciences, Nanyang Technological University, Singapore, 637371 Singapore; 4 Department of Environment Management, Indian Institute of Social Welfare and Business Management, Kolkata, 700073 India

**Keywords:** Platinum group element (PGE), Catalytic converters, ICP-MS, PM_10_ and PM_2.5_, Monsoon and winter

## Abstract

This study investigates platinum group elements (PGEs) in the breathable (PM_10_) and respirable (PM_2.5_) fractions of air particulates from a heavily polluted Indian metro city. The samples were collected from traffic junctions at the heart of the city and industrial sites in the suburbs during winter and monsoon seasons of 2013–2014. PGE concentrations were determined by inductively coupled plasma-mass spectrometry (ICP-MS). The PGE concentrations in the samples from traffic junctions are within the range of 2.7–111 ng/m^3^ for Pd, 0.86–12.3 ng/m^3^ for Pt and 0.09–3.13 ng/m^3^ for Rh, and from industrial sites are within the range of 3.12–32.3 ng/m^3^ for Pd, 0.73–7.39 ng/m^3^ for Pt and 0.1–0.69 ng/m^3^ for Rh. Pt concentrations were lower in the monsoon compared to winter while Pd concentrations increased during monsoon and Rh stayed relatively unaffected across seasons. For all seasons and locations, concentrations of Pd > Pt > Rh, indicating dominance of Pd-containing exhaust converters. Most of the PGEs were concentrated in the PM_2.5_ fraction. A strong correlation (R ≥ 0.62) between the PGEs from traffic junction indicates a common emission source viz. catalytic converters, whereas a moderate to weak correlation (R ≤ 0.5) from the industrial sites indicate mixing of different sources like coal, raw materials used in the factories and automobile. A wider range of Pt/Pd, Pt/Rh and Pd/Rh ratios measured in the traffic junction possibly hint towards varying proportions of PGEs used for catalyst productions in numerous rising and established car brands.

**Electronic supplementary material:**

The online version of this article (doi:10.1186/s40064-016-2854-5) contains supplementary material, which is available to authorized users.

## Background

Platinum group elements (PGE), in particular platinum (Pt), palladium (Pd), and rhodium (Rh), are few of the least abundant elements in the Earth’s continental crust, with estimated concentrations of 0.4–0.06 ppb (Wedepohl 1995). These metals and their compounds are highly valued by chemical, food and pharmaceutical industries for use as catalysts in a wide range of reactions like hydrogenation, hydrogenolysis, coupling reaction, etc. Automobile catalytic converters that used Pt and Pd were first introduced in the US in the mid-1970s to curb the harmful emissions of nitrogen oxide (NO_x_), carbon monoxide and polycyclic aromatic hydrocarbons (PAHs) from automobile exhaust. The 1970s Pt–Pd catalytic converters gradually evolved to the present day three-way automobile catalytic converters that utilize Pt, Pd and Rh (Cicchella et al. 2003; Zereini et al. 2007). Present day global consumption of Pt, Pd and Rh are dominated by the automobile catalytic converter industry accounting for 37, 72 and 79 % of the global demand respectively (JMPLC 1999, 2013).

Studies on the impacts of these noble elements have grown in prominence since 1990s due to evidence of anthropogenic emissions from autocatalytic converters leading to elevated levels of these elements in the environment (e.g. Zereini et al. 1997, 2001, 2004, 2007; Rauch et al. 2001, 2005, 2006; Gomez et al. 2002; Pan et al. 2009; Bozlaker et al. 2014). PGE emissions from catalysts are linked to mechanical abrasion and highly variable redox chemical conditions during engine operation (Palacios et al. 2000; Moldovan et al. 2002). Other known anthropogenic PGE fluxes to the environment include coal combustion, noble metal production and medical treatment (Zereini et al. 2012; Rauch and Peucker-Ehrenbrink 2015). After deposition, PGE could be subjected to various physical and chemical transformations, potentially resulting in migration into other environmental compartments such as the biota (Morton-Bermea et al. 2014).

There has been growing concern regarding the widespread dispersion of PGE in the troposphere of the Northern Hemisphere. For example, Greenland snow fall from the mid 1990s had approximately 40–120 times higher PGE concentration compared to ice from 7000 years ago (Barbante et al. 2001). Several studies have also reported elevated levels of PGE in airborne PM (e.g. Gomez et al. 2002; Kantisar et al. 2003; Zereini et al. 2004; Limbeck et al. 2004; Rauch et al. 2006).

Growing evidence indicates short-term and possibly long-term adverse impacts of PGE and its compounds on human health. The most significant health effect caused by exposure to soluble PGE compounds is sensitization. Many studies had reported cases of occupational allergic contact dermatitis, asthma, urticarial, rhinitis and conjunctivitis (Schierl and Ochmann 2015; Wiseman, 2015). To understand the biogeochemical cycling of the PGEs, more studies focusing on sources, pathways, sinks and post depositional uptake by biota are required. Many studies conducted thus far are focused mainly on the vehicular emission of PGE in the environment. However industrial emissions can also contribute towards an elevated level of PGE in the atmospheric PM.

We sampled air particulates from in and around Kolkata, a megacity in Eastern India. The trace metal composition of PM_10_ and PM_2.5_ apportioned three primary anthropogenic sources, vehicular exhausts in the city traffic junctions and coal combustion and high temperature metal smelting in the suburban industrial areas (Das et al. 2015). We also measured PGEs from these locations. For this report we divided the sampling locations into two categories; traffic junctions in the heart of the city and industrial hubs in the city suburbs. The purpose of the study is to access industrial contribution of PGE fluxes in addition to automobile emission and the effect of seasonal changes on the distribution of PGE in air particulate.

## Methods

### Study area

Kolkata, the state capital of West Bengal in India, is located at approximately 22.6°N, 88.4°E. The city has a population of approximately 4 million (Census of India 2011), making it the third-most populous metropolitan area in India. Located on the east bank of the River Hughli, the city sits on alluvium within the lower Ganges Delta. This results in a relatively flat landscape, with an average elevation of the city about 6.4 m above mean sea level. The climate of the city is tropical savanna under the Köppen climate classification with total annual rainfall of 1582 mm. As Kolkata is located at a point where River Hughli merges into the Bay of Bengal, the sea is a major influence in the weather pattern of the city. The southwest summer Monsoon, caused by tropical depressions in the Bay of Bengal, hits Kolkata between June and September. The winter in the city is relatively dry and lasts for about 2.5 months from late November to January.

### Sampling

Sampling was carried out between 6 AM and 6 PM with two Deployable Particulate Sampler (DPS) pumps (Leland Legacy) with a pumping efficiency of 10 L/min. The two pumps were used to collect the PM_10_ and PM_2.5_ simultaneously. The PM was collected on 47 mm quartz filters that were dried overnight at 400 °C in an oven prior to sampling.

We conducted sampling in two seasons in six traffic junctions inside the city of Kolkata and 6 industrial sites in the suburbs where road traffic is far less compared to the city (Additional file 1: Figure S1). The six industrial sites include two coal fired thermal power plant, two cement industry and two industrial estates comprising of various industries including electronics, textile, food processing, plastic/rubber, chemical, leather goods, iron ore etc. To mitigate the effect of long-range transport of suspended particulate matters from the roads in the urban area to the suburban industrial areas, the pumps in the industrial sites were placed in close proximity (several hundred meters) of the exhaust fumes emitted from the factories.

Sampling was carried out in winter of 2013–2014 (December–January) and in monsoon season of 2014 (August–September). During the winter sampling, the daily average temperature ranged from 15 to 23 °C, relative humidity varied between 59 and 82 % and the predominant wind direction was N to NNW. During the monsoon sampling season daily average temperature was 24–32 °C, relative humidity 74–93 % and wind direction was S to SW. Several previous studies (Chowdhury 2004; Spiroska et al. 2011; WBPCB 2012) in different Indian metropolises reported highest pollutant levels during the cooler months as the inversion layer remains close to the ground and lowest pollutant levels during the monsoon. Hence we choose to carry out our sampling during the winter and monsoon months in Kolkata to capture the largest possible seasonal variation in PGE concentration in the air particulate, if present.

### Chemical analysis

The filters were placed in Teflon vessels and subjected to microwave digestion in a mixture of ultra-high purity acids (6 mL HNO_3_, 2 mL HCl and 4 mL ultrapure water). The microwave (Milestone ETHOS, Italy) temperature was ramped to 160 °C over 15 min and held in this temperature for 10 min prior to a 30 min cooling period. Complete solubilization of PGE was achieved with this digestion procedure. After cooling the digested samples were diluted with ultra-pure water, filtered, transferred to polyethylene bottles and then stored in the fridge for ICP-MS analysis.

Prepared samples were analyzed for PGE using an Agilent 7700 series Inductively Coupled Plasma-Mass Spectrometer (Japan) equipped with a 3rd generation He reaction/collision cell (ORS^3^) to minimize interferences. The operating conditions used for the analysis of samples are shown in Additional file 1: Table S1. To validate both digestion and ICP-MS method, NIST SRM 2783 (Air Particulate on Filter Media) was measured and blank filters were spiked with Pd, Pt and Rh standards and treated in the same way as the samples. Recoveries from these spiked filters ranged from 82 to 90 %.

## Results and discussion

**Fig. 1:**
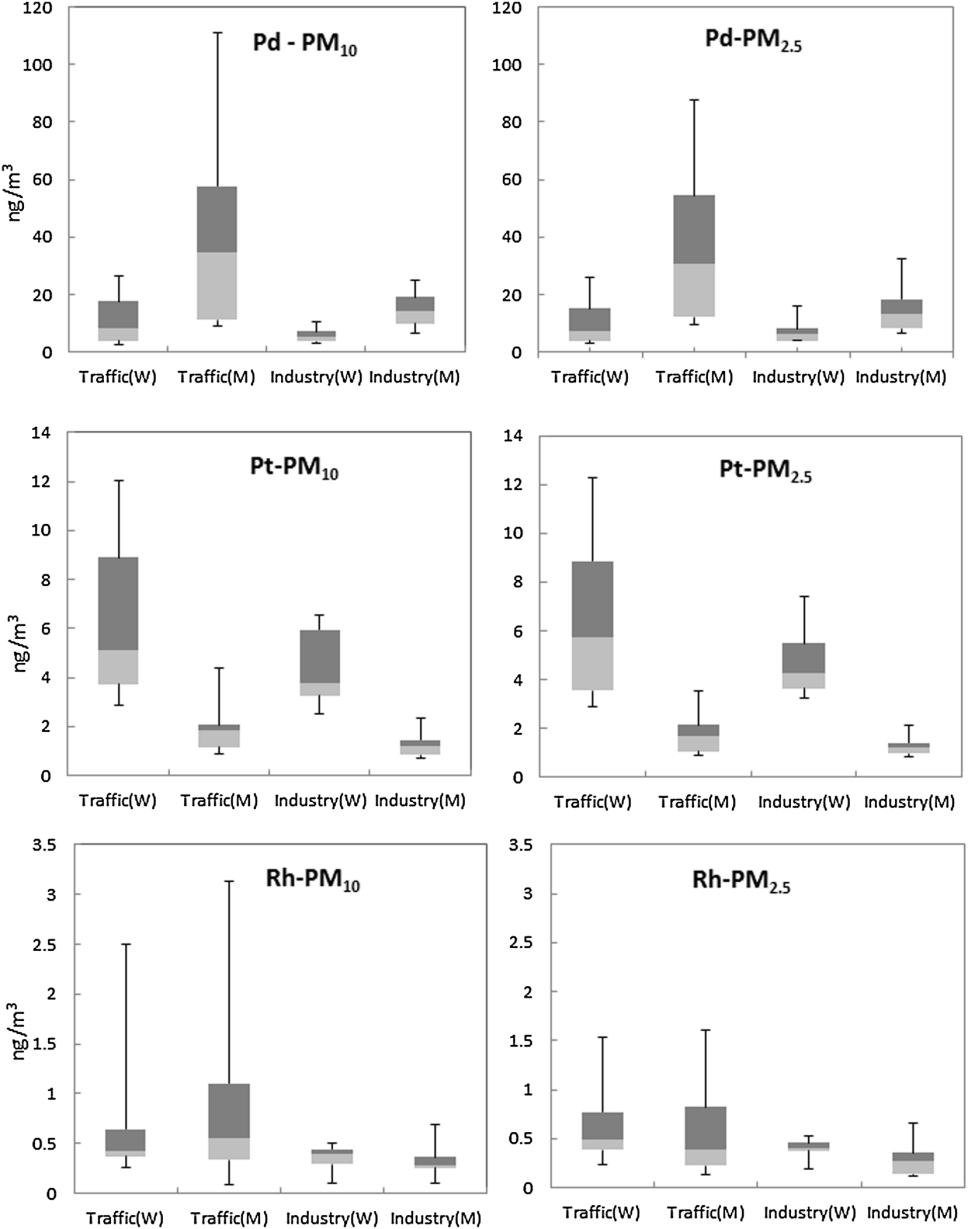
A comparison of PGE concentrations in PM_10_ and PM_2.5_ during winter (W) and monsoon (M) seasons from traffic and industrial locations

PGE concentrations in the samples collated from industrial areas and traffic junctions for both winter and monsoon seasons in Kolkata show elevated values as compared to continental crust (Pd: 0.4 ng/g; Pt: 0.4 ng/g; Rh: 0.06 ng/g, Wedepohl 1995). The observed mean PM_10_ concentrations of Pd, Pt and Rh in industrial areas during winter are 5.94, 4.37 and 0.35 ng/m^3^ respectively while measured concentrations at traffic junctions average 10.8, 6.27 and 0.70 ng/m^3^ respectively. For the monsoon season mean PM_10_ concentrations of Pd, Pt and Rh in the roadside traffic junctions are 41, 1.92 and 0.88 ng/m^3^ respectively whereas those at the industrial sites average 14.6, 1.25 and 0.31 ng/m^3^ respectively. The mean PM_2.5_ concentrations of Pd, Pt and Rh in traffic junctions during winter are 9.79, 6.45 and 0.62 ng/m^3^ respectively while those at industrial sites average 6.97, 4.68 and 0.40 ng/m^3^ respectively. In the monsoon season the Pd, Pt and Rh in traffic junctions were recorded to be 36.2, 1.76 and 0.59 ng/m^3^ respectively and from the industrial areas 14.4, 1.22 and 0.28 ng/m^3^ respectively (Table 1; Fig. 1).

**Table 1:** Mean PM_10_ and PM_2.5_ concentrations and PGE concentrations (min–max) in PM_10_ and PM_2.5_ from traffic junctions and industrial locations in and around Kolkata, India compared with dust and soil concentrations from the city

	N	PM Concentration (µg/m^3^)	Pd (ng/m^3^)	Pt (ng/m^3^)	Rh (ng/m^3^)
*PM*_*10*_					
Traffic					
Winter	20	385 (78–783)	10.8 (2.71–26.4)	6.27 (2.85–12.1)	0.70 (0.26–2.50)
Industry					
Winter	15	392 (61–767)	5.94 (3.12–10.3)	4.37 (2.53–6.53)	0.35 (0.10–0.50)
Traffic					
Monsoon	14	276 (83–411)	41 (9.10–110.94)	1.92 (0.90–4.39)	0.88 (0.09–3.13)
Industry					
Monsoon	14	302 (83–617)	14.6 (6.50–16.4)	1.25 (0.73–2.35)	0.31 (0.10–0.69)
*PM*_*2.5*_					
Traffic					
Winter	20	316 (83–489)	9.79 (2.87–25.9)	6.45 (2.90–12.3)	0.62 (0.23–1.53)
Industry					
Winter	15	271 (167–583)	6.97 (3.81–15.8)	4.68 (3.26–7.39)	0.40 (0.19–0.53)
Traffic					
Monsoon	14	117 (44–178)	36.2 (9.39–87.8)	1.76 (0.86–3.54)	0.59 (0.13–1.61)
Industry					
Monsoon	14	264 (117–578)	14.4 (6.76–32.3)	1.22 (0.82–2.10)	0.28 (0.12–0.66)
Dust^a^					
Kolkata	10		10.8 (0.7–63.6)	13.5 (2.6–51)	3.6 (0.2–21.3)
Soil^b^					
Kolkata	3		2.83 (1.31–4.07)	5.59 (2.59–9.43)	1.03 (0.40–2.27)

^a^Pan et al. (2013), ^b^ Pan et al. (2009)

Clearly, most of the emitted PGEs from catalytic converters and/or coal combustion are associated with finer PM size fractions that are readily trapped by the alveoli of the lungs and can cause negative respiratory health effects in exposed populations. Pd makes up the largest fraction of PGE contribution as compared to Rh and Pt, regardless of seasons and sampling location. Traffic junction sites have higher PGE concentrations compared to industrial areas (Fig. 1) for both size fractions. Pd concentrations are significantly higher in PM_10_ and PM_2.5_ in monsoon season compared to winter whereas Pt concentration decreases in monsoon and Rh concentration stays constant.

### PGE concentrations in air particulate

Most of the previous studies had mainly focused on vehicle emission of PGE from the catalytic converters (Pan et al. 2009; Gao et al. 2012; Palacios et al. 2000; Zereini et al. 2012; Wichmann and Bahadir 2015). Only few studies had highlighted the importance of industrial activities and coal combustion in assessing urban PGE fluxes (Liu et al. 2015; Rauch and Peucker-Ehrenbrink 2015). After a ruling by the Supreme Court in 1995, all new petrol-fueled cars in India were to be fitted with catalytic converters. Hence, a major source of PGE in the urban PM of India is road traffic. However, traffic is not the sole source of PGE in urban atmosphere as several studies confirmed the enrichment of Pt and Pd in coal though the absolute concentrations would depend on different regional distribution of coal resources (Dai et al. 2003; Wang et al. 2008). Kolkata suburbs have numerous industries including coal fired thermal power stations, numerous brick kilns along the banks of River Hughli, cement factories, medium and small scale industrial estates, etc.

The observed concentration trend of Pd > Pt > Rh in both the traffic and industrial locations matches the PGE trend reported in other recent studies (Gao et al. 2012; Liu et al. 2015). However, when compared to other megacities around the world, the PGE concentrations measured in Kolkata are orders of magnitude higher for both the locations and in both the size fractions (Table 2). The PGE concentrations measured in the present study matches rainfall concentrations (average concentrations of Pd is 26.73 ng/L; Pt is 1.71 ng/L and Rh is 1.49 ng/L) measured in downtown Changji City in Xinjiang, China (Liu et al. 2015). Previous studies (Pan et al. 2009, 2013) on PGE concentrations in dust and soil samples from Kolkata shows comparable concentration ranges (Table 1).

**Table 2:** A comparison of PGE concentrations in PM_10_ and PM_2.5_ from various cities around the world

City, Country	PM	Sampling period	Pd (ng/m^3^)	Pt (ng/m^3^)	Rh (ng/m^3^)	References
Boston, Massacheutts, USA	10	2002–2003	0.009 (0.0008–0.039)	0.0078 (0.006–0.036)	0.0019 (0.00033–0.0059)	Rauch et al. (2005)
Mexico City, Mexico	10	2003	0.011	0.0093	0.0032	Rauch et al. (2006)
Beijing, China	10	Oct–Dec 2007		0.0218 (0.00622–0.0243)	0.00744 (0.00116–0.0860)	Pan et al. (2009)
Guangzhou, China				0.0109 (0.00768–0.0122)	0.00468 (0.00215–0.00515)	
Raleigh, North Carolina, USA	10	Oct–Dec 2006	0.029	0.020	0.001	Hays et al. (2011)
Frankfurt am Main, Germany	10	2009–2010	0.058 (0.008–0.132)	0.067 (0.006–0.135)	0.013 (0.002–0.035)	Zereini et al. (2012)
Vienna, Austria	10	2009–2011	0.0106 (0.0043–0.0216)	0.0099 (0.0048–0.0185)		Puls et al. (2012)
Texas, USA	10	2012–2013	0.214 (0.1304–0.2986)	0.0611 (0.0382–0.0840)	0.0363 (0.0212–0.0515)	Bozlaker et al. (2014)
Braunschweig, Germany	10	2005	0.0378 (0.0001–0.044)	0.159 (0.0009–0.246)	0.0010 (0.0002–0.015)	Wichmann and Bahadir (2015)
Frankfurt am Main, Germany	2.5	2009–2010	0.0161 (0.0054–0.0274)	0.0094 (0.0026–0.0236)	0.0018 (0.007–0.0042)	Zereini et al. (2012)
Vienna, Austria	2.5	2009–2011	0.0049 (<LOD–0.0097)	0.0023 (<LOD–0.0046)		Puls et al. (2012)
Texas, USA	2.5	2012–2013	0.0911 (0.0709, 0.113)	0.0301 (0.0220, 0.0382)	0.0125 (0.0085, 0.0166)	Bozlaker et al. (2014)
Istanbul, Turkey	2.5	2010–2011	0.00042 (0.00022–0.00064)			Atilgan et al. (2012)
Budapest, Hungary	2.5	2010–2011	0.00048 (0.00025–0.00086)			Atilgan et al. (2012)

Numbers in parenthesis indicate concentration range.

*LOD* limit of detection

PGE concentrations measured from industrial areas and power plants in this study have found high Pd, Pt and Rh concentrations, however the total concentrations are less than those measured at traffic junctions in both the seasons. This reinforces the suggestion by few studies (Liu et al. 2015; Rauch and Peucker-Ehrenbrink 2015) that industrial emissions play an equally important role as automobile emissions in PGE fluxes in the environment.

Correlation analysis conducted for traffic areas and industrial areas suggests that PGE fluxes in industrial areas have multiple sources. A strong correlation between the concentrations of two elements, as indicated by large R-values, hints a common source of emission. As summarized in Table 3, strong correlation (R-value > 0.6) for Pt/Pd, Pt/Rh and Pd/Rh found in traffic areas, regardless of PM sizes and seasons, imply that PGE fluxes in traffic areas are contributed mainly by the same source, vehicle emission. Conversely, relatively weak correlations between Pd, Pt and Rh in the industrial areas probably imply mixing of multiple sources, coal combustion, raw materials used in different factories and vehicle emission.

**Table 3:** Correlation coefficients of PGEs in traffic junctions and industrial areas

	Traffic				Industrial			
	PM_10_		PM_2.5_		PM_10_		PM_2.5_	
	Winter	Monsoon	Winter	Monsoon	Winter	Monsoon	Winter	Monsoon
Pt/Pd	0.79	0.82	0.77	0.63	0.04	0.38	0.06	0.35
Pt/Rh	0.64	0.93	0.89	0.81	0.50	0.19	0.19	0.30
Pd/Rh	0.62	0.80	0.89	0.74	0.14	0.50	0.24	0.29

### PGE ratios as source indicators

Pt/Pd, Pt/Rh and Pd/Rh ratios are calculated for different seasons and different particulate sizes (Table 4). The PGE ratios (Pt/Pd, Pt/Rh and Pd/Rh) in PM_10_ in industrial area during the winter season are 0.81, 13.6 and 22.2 while for the traffic junctions in the main city area are 0.74, 10.8 and 17.0 respectively. PGE ratio values obtained for PM_2.5_ are 0.77, 12.3 and 20.1 in the industrial areas and 0.81, 10.9 and 15.5 in the traffic junctions respectively. A comparison of Pt/Pd, Pt/Rh and Pd/Rh mean ratios between traffic and industrial areas could not conclusively determine whether PGE emissions originates from catalytic converter or other potential sources (p > 0.05). Recent studies (Pan et al. 2009; Qi et al. 2011; Zereini et al. 2012; Gao et al. 2012; Liu et al. 2015) have revealed wider PGE ratio ranges compared to older studies, irrespective of regions. The PGE components in catalytic converter are constantly changing to optimize its efficiency and function. A shift towards larger Pd/Rh and smaller Pt/Pd ratios implies higher Pd concentrations present in the environment due to increased usage. In this study, higher Pd concentration found in the traffic area could be explained by the switch from Pt-dominant catalytic converter to Pd-containing exhaust converter (Zereini et al. 2012). The shift towards using Pd over Pt converters is a cause of concern, as Pd poses a greater risk to human health due to its greater solubility and hence bioavailability (Colombo et al. 2008a, b; Wiseman and Zereini 2009).

**Table 4:** PGE ratios from various cities in the world

City, Country	Sample	Pt/Pd	Pt/Rh	Pd/Rh
Indiana, US^a^	–	1–2.5	5–16	4–9
Perth, Australia^b^	Road dust	1.04 (0.7–1.5)	5.1 (4.6–6.3)	5.17 (3.3–6.9)
	Roadside soil	1.4	6.1	4.5
Boston, US^c^	Airborne particles	1.01	5.06	
Mexico City, Mexico^d^	Airborne particles	0.96	3.1	
Vienna, Austria^e^	Airborne particles	3.41		
Beijing,China^f^	Roadside soil	1.91* (0.13–3.82)	3.94 (2.40–19.6)	2.06* (1.83–31.3)
Guangzhou, China^f^		0.88*	2.9*	3.3*
Hong Kong, China^f^		1.61*	5.76*	3.58*
Macao, China^f^		0.88*	4.21*	4.8*
Qingdao, China^f^		1.0*	3.23*	3.24*
Mumbai, India^f^		0.4*	9.75*	24.2*
Kolkata, India^f^		1.98*	5.43*	2.75*
Shenzhen, China^g^	Road dust and roadside soil	0.93 (0.35–2.17)	3.96 (2.87–10.8)	
Hong Kong, China^g^		0.94 (0.3–1.98)	8.51 (4.4–14.9)	
Guangzhou, China^g^		0.51 (0.21–1.35)	3.37 (1.49–5.62)	
Frankfurt, Germany^h^	Airborne particles	(0.04–5.0)*	3.8 (0.3–19.2)	
Beijing, China^i^	Road dust	0.59 (0.2–3.2)*	(1.0–5.8)*	
Xinjiang, China^j^	Rainwater			17.94
Kolkata, India (current study)	PM_10_ winter			
	Traffic	0.74 (0.38–1.20)	10.8 (4.0–21.0)	17.0 (8.87–47.5)
	Industrial	0.81 (0.35–1.19)	13.6 (8.08–25.3)	22.2 (8.64–68.5)
	PM_2.5_ winter			
	Traffic	0.81 (0.36–1.91)	10.9 (5.73–20.5)	15.5 (8.54–38.5)
	Industrial	0.77 (0.27–1.05)	12.3 (8.46–27.7)	20.1 (9.0–58.7)

^a^ Ely et al. (2001), ^b^ Whiteley and Murray (2003), ^c^ Rauch et al. (2005), ^d^ Rauch et al. (2006), ^e^ Limbeck et al. (2007), ^f^ Pan et al. (2009), ^g^ Qi et al. (2011), ^h^ Zereini et al. (2012), ^i^ Gao et al. (2012), ^j^ Liu et al. (2015)

* Author’s estimation from available data

Larger PGE ratio range observed in traffic area may be attributed to varying proportions of PGE used for catalyst productions in many rising and established car brands. Aided by the strengthening of economies in developing countries like India and China, the use of personalized cars has been rapidly increasing. In addition, new vehicles purchased in Kolkata since April 2005 would be fitted with catalytic converter due to the implementation of Euro III emission standards in 11 metro cities by the Indian government (Pan et al. 2009). In short, an increase in the number of new cars fitted with catalytic converter of numerous competing car brands in the market may have resulted in the wide PGE ratio observed in this study.

Wide-ranging PGE ratio found in industrial areas could be rationalized by the fact that different raw materials with wide ranging PGE ratios are used by various industries for their operations. For example 122 coal samples measured from USA had Pt/Pd ratio ranging from 0.37 to 2.22, Pt/Rh ratio from 1 to 10.9 and Pd/Rh ratio from 1 to 8.5 (Oman et al. 1997). Hence to better assess the individual contribution of raw materials from these industries, further studies are required to establish PGE ratio ranges of raw materials particularly coal used by different industries.

### Seasonal variation of PGE concentrations

An assessment of Pd, Pt and Rh concentration trend (Fig. 1) between winter and monsoon seasons reveals an interesting trend. Several studies have observed a higher Pd and Pt concentration in airborne PM (Zereini et al. 2012; Rauch et al. 2005) during dry or winter season as compared to other seasons. Scavenging of aerosols by rain was suggested as a probable reason for this trend (Zereini et al. 2012). Other possible cause could be due to thermal inversions that dominate during winter, leading to the accumulation of aerosol in the atmosphere.

However, in our current study, spikes in the mean PM_10_ concentration of Pd by 146 and 280 % were observed during monsoon season as compared to winter for industrial and traffics areas respectively (Fig. 1). Similar magnitude of increase was also observed for PM_2.5_ in both locations. On the other hand, a noticeable decrease in Pt concentration during monsoon by approximately 70 % for both PM_10_ and PM_2.5_ were observed which we attribute to the scavenging effect of the rain. Average concentrations of Rh for PM_10_ and PM_2.5_ were relatively unaffected in both seasons.

We hypothesize the trend in our monsoon data to be due to interplay of scavenging effect of rain and the solubility of Pd, Pt and Rh elements or their compounds in moist air. Washout of aerosol by rain can explain the decrease in Pt concentration during monsoon. Rh is least soluble in water which justifies its near constant concentrations across seasons. We hypothesize the increase of Pd concentration during monsoon is due to higher solubility of Pd and its various species in the hygroscopic aerosols during monsoon (Jarvis et al. 2001; Whiteley and Murray 2003). Due to the high humidity in Kolkata (average ~87 % during sampling season) the hydrated aerosols dissolve Pd emitted into the atmosphere from vehicle and industrial sources and lengthen its residence time in the atmosphere. As measurements are conducted in two short periods during winter and monsoon seasons, a longer period of monitoring could test our hypothesis and assist in establishing PGE trend in the atmosphere in various seasons.

## Conclusion

In India, PGE data archives in environmental samples are inadequate, particularly after the year 2000 when passenger cars and commercial vehicles were required to meet the emission level of Euro I standard. Post 2005, new cars in the Indian metro cities have had to meet the emission standards equivalent to Euro III. Measurements conducted in Kolkata from traffic junctions and industrial sites show high concentration of PGE in the air and a trend of Pd > Pt > Rh during winter and monsoon seasons in both the locations. A strong correlation could be found for Pt/Pd, Pt/Rh and Pd/Rh in traffic areas during both winter and monsoon seasons, which indicate a common emission source (i.e. automobile catalytic converter). Conversely, weak or moderate correlations are observed for Pt/Pd, Pt/Rh and Pd/Rh, which we attribute to various distinctive PGE ratios in different raw materials used by industries. Our study could not conclusively pinpoint industrial or traffic PGE emission based on PGE ratios. We noticed a wide ratio range for Pt/Pd, Pt/Rh and Pd/Rh in contrary to previous studies. Seasonal variations in atmospheric PGE concentration were observed. During monsoon season concentrations of Pd increases by 146 and 280 % in industrial and traffic areas respectively whereas Pt dropped by approximately 70 % for both locations. As measurements are conducted in two short periods during winter and monsoon seasons, a longer period of monitoring could assist in establishing PGE trends in the atmosphere due to changing seasons. Further studies are required to establish PGE ratio range for different industrial raw materials and emissions to assess their individual contribution to PGE flux in the atmosphere.

## Additional file

10.1186/s40064-016-2475-z Agilent 7700 ICP-MS operating conditions for the analysis of PM samples. **Figure S1.** Map showing sampling locations in and around the megacity of Kolkata, India.

## References

[CR1] Atilgan S, Akman S, Baysal A, Bakircioglu Y, Szigeti T, Ovari M, Zaray G (2012). Monitoring of Pd in airborne particulates by solid sampling high-resolution continuum source electrothermal atomic absorption spectrometry. Spectrochim Acta, Part B.

[CR2] Barbante C, Veysseyre A, Ferrari C, Van de Velde K, Morel C, Capodaglio G, Cescon P, Scarponi G, Boutron C (2001). Greenland snow evidence of large scale atmospheric contamination for platinum, palladium, and rhodium. Environ Sci Technol.

[CR3] Bozlaker A, Spada NJ, Fraser MP, Chellam S (2014). Elemental characterization of PM2.5 and PM10 emitted from light duty vehicles in the Washburn Tunnel of Houston, Texas: release of rhodium, palladium, and platinum. Environ Sci Technol.

[CR4] Census of India, Office of the Register General & Census Commissioner (2011) Ministry of Home Affairs, India. http://censusindia.gov.in/2011-prov-results/prov_data_products_wb.html. Accessed 6 July 2015

[CR5] Chowdhury MZ (2004) Characterization of fine particle air pollution in the Indian subcontinent. Ph.D. Thesis, Georgia Institute of Technology

[CR6] Cicchella D, De Vivo B, Lima A (2003). Palladium and platinum concentration in soils from the Napoli metropolitan area, Italy: possible effects of catalytic exhausts. Sci Total Environ.

[CR7] Colombo C, Monhemius AJ, Plant JA (2008). Platinum, palladium and rhodium release from vehicle exhaust catalysts and road dust exposed to simulated lung fluids. Ecotoxicol Environ Saf.

[CR8] Colombo C, Monhemius AJ, Plant JA (2008). The estimation of the bioavailabilities of platinum, palladium and rhodium in vehicle exhaust catalysts and road dusts using a physiologically based extraction test. Sci Total Environ.

[CR9] Dai S, Ren D, Zhang J, Hou X (2003). Concentrations and origins of platinum group elements in Late Paleozoic coals of China. Int J Coal Geol.

[CR10] Das R, Khezri B, Srivastava B, Datta S, Sikdar PK, Webster RD, Wang X (2015). Trace Element Composition of PM2.5 and PM10 from Kolkata—a Heavily Polluted Indian Metropolis. Atmos Pollut Res.

[CR11] Ely JC, Neal CR, Kulpa CF, Schneegurt MA, Seidler JA, Jain JC (2001). Implications of platinum-group element accumulation along U.S. Roads from Catalytic-Converter Attrition. Environ Sci Technol.

[CR12] Gao B, Yu Y, Zhou H, Lu J (2012). Accumulation and distribution characteristics of platinum group elements in roadside dusts in Beijing, China. Environ Toxicol Chem.

[CR13] Gomez B, Palacios MA, Gomez M, Sanchez JL, Morrison G, Rauch S, McLeod C, Ma R, Caroli S, Alimonti A, Petrucci F, Bocca B, Schramel P, Zischka M, Petterson C, Wass U (2002). Levels and risk assessment for humans and ecosystems of platinum-group elements in the airborne particles and road dust of some European cities. Sci Total Environ.

[CR14] Hays MD, Cho SH, Baldauf R, Schauer JJ, Shafer M (2011). Particle size distributions of metal and non-metal elements in an urban near-highway environment. Atmos Environ.

[CR15] Jarvis KE, Parry SJ, Piper JM (2001). Temporal and spatial studies of autocatalyst-derived platinum, rhodium, and palladium and selected vehicle-derived trace elements in the environment. Environ Sci Technol.

[CR16] JMPLC (Johnson Matthey Public Limited Company) (1999) Platinum 1999 interim review. http://www.platinum.matthey.com/services/market-research/market-review-archive/platinum-1999-interim-review. Accessed 6 July 2015

[CR17] JMPLC (Johnson Matthey Public Limited Company) (2013) Platinum 2013 interim review. http://www.platinum.matthey.com/services/market-research/market-review-archive/platinum-2013-interim-review. Accessed 6 July 2015

[CR18] Kantisar K, Koellensperger G, Hann S, Limbeck A, Puxbaum H, Stingeder G (2003). Determination of Pt, Pd, and Rh by inductively coupled plasma sector field mass spectroscopy (ICP-SFMS) in size-classified urban aerosol samples. J Anal At Spectrom.

[CR19] Limbeck A, Rendl J, Heimburger G, Kranabetter A, Puxbaum H (2004). Seasonal variation of palladium, elemental carbon and aerosol mass concentrations in airborne particulate matter. Atmos Environ.

[CR20] Limbeck A, Puls C, Handler M (2007). Platinum and palladium emissions from on-road vehicles in the Kaisermühlen Tunnel (Vienna, Austria). Environ Sci Technol.

[CR21] Liu Y, Tian F, Liu C, Zhang L (2015). Platinum group elements in the precipitation of the dry region of Xinjiang and factors affecting their deposition to land: the case of Changji City, China. Atmos Pollut Res.

[CR22] Moldovan M, Palacios MA, Gómez MM, Morrison G, Rauch S, McLeod C, Ma R, Caroli S, Alimonti A, Petrucci F, Bocca B, Schramel P, Zischka M, Pettersson C, Wass U, Luna M, Saenz JC, Santamarıa J (2002). Environmental risk of particulate and soluble platinum group elements released from gasoline and diesel engine catalytic converters. Sci Total Environ.

[CR23] Morton-Bermea O, Amador-Muñoz O, Martínez-Trejo L, Hernández-Álvarez E, Beramendi-Orosco L, García-Arreola ME (2014). Platinum in PM2.5 of the metropolitan area of Mexico City. Environ Geochem Hlth.

[CR24] Oman CL, Finkelman RB, Tewalt SJ (1997) Concentrations of platinum group elements in 122 U.S coal samples. In: U.S. Geological Survey Open-File Report 97-53. U.S. Geological Survey. http://pubs.usgs.gov/of/1997/of97-053/. Accessed 3 July 2015

[CR25] Palacios MA, Gomez MM, Moldovan M, Morrison G, Rauch S, McLeod C, Ma R, Laserna J, Lucena P, Caroli S, Alimonti A, Petrucci F, Bocca B, Schramel P, Lustig S, Zischka M, Wass U, Stenbom B, Luna M, Saenz JC, Santamaria J (2000). Platinum-group elements: quantification in collected exhaust fumes and studies of catalyst surfaces. Sci Total Environ.

[CR26] Pan S, Zhang G, Sun Y, Chakraborty P (2009). Accumulating characteristics of platinum group elements (PGE) in urban environments, China. Sci Total Environ.

[CR27] Pan S, Sun Y, Zhang G, Chakraborty P (2013). Spatial distributions and characteristics of platinum group elements (PGEs) in urban dusts from China and India. J Geochem Explor.

[CR28] Puls C, Limbeck A, Hann S (2012). Bioaccessibility of palladium and platinum in urban aerosol particulates. Atmos Environ.

[CR29] Qi L, Zhou M, Zhao Z, Hu J, Huang Y (2011). The characteristics of automobile catalyst-derived platinum group elements in road dusts and roadside soils: a case study in the Pearl River Delta region, South China. Environ Earth Sci.

[CR30] Rauch S, Peucker-Ehrenbrink B (2015) Sources of platinum group elements in the environment. In: Zereini F, Wiseman CLS (Eds) Platinum metals in the environment. Springer, Heidelberg, pp 3–17

[CR31] Rauch S, Lu M, Morrison GM (2001). Heterogeneity of Platinum group metals in airborne particles. Environ Sci Technol.

[CR32] Rauch S, Hemond HF, Peucker-Ehrenbrink B, Ek KH, Morrison GM (2005). Platinum group element concentrations and osmium isotopic composition in urban airborne particles from Boston, MA. Environ Sci Technol.

[CR33] Rauch S, Peucker-Ehrenbrink B, Molina LT, Molina MJ, Ramos R, Hemond HF (2006). Platinum group elements in airborne particles in Mexico City. Environ Sci Technol.

[CR34] Schierl R, Ochmann U (2015) Occupational health aspects of platinum. In: Zereini F, Wiseman CLS (eds) Platinum metals in the environment. Springer, Heidelberg, pp 463–476

[CR35] Spiroska J, Rahman MA, Pal S (2011). Air pollution in Kolkata: an analysis of current status and interrelation between different factors. SEEU Rev.

[CR36] Wang H, Zhu YM, Zhao WY (2008). The geological researched status of platinum–group elements in coal. Geol Sci Technol Info.

[CR37] Wedepohl KH (1995). The composition of the continental crust. Geochim Cosmochim Acta.

[CR38] West Bengal Pollution Control Board (WBPCB) (2012) A report on trend of important air quality parameters in kolkata during night time as compared to daytime situation during year 2011 and 2012. West Bengal Pollution Control Board (WBPCB), Kolkata http://emis.wbpcb.gov.in/airquality/daynightreport.do. Accessed 20 June 2015

[CR39] Whiteley JD, Murray F (2003). Anthropogenic platinum group element (Pt, Pd and Rh) concentrations in road dusts and roadside soils from Perth, Western Australia. Sci Total Environ.

[CR40] Wichmann H, Bahadir M (2015) Increase of platinum group element concentrations in soils and airborne dust during the period of vehicular exhaust catalysts introduction. In: Zereini F, Wiseman CLS (eds) Platinum metals in the environment. Springer, Heidelberg, pp 153–161

[CR41] Wiseman CLS (2015) Platinum metals in airborne particulate matter and their bioaccessibility. In: Zereini F, Wiseman CLS (eds) Platinum metals in the environment. Springer, Heidelberg, pp 447–462

[CR42] Wiseman CLS, Zereini F (2009). Airborne particulate matter, platinum group elements and human health: a review of recent evidence. Sci Total Environ.

[CR43] Zereini F, Skerstupp B, Alt F, Helmers E, Urban H (1997). Geochemical behaviour of platinum-group elements (PGE) in particulate emissions by automobile exhaust catalysts: experimental results and environmental investigations. Sci Total Environ.

[CR44] Zereini F, Wiseman C, Alt F, Messerschmidt J, Müller J, Urban H (2001). Platinum and rhodium concentrations in airborne particulate matter in Germany from 1988 to 1998. Environ Sci Technol.

[CR45] Zereini F, Alt F, Messerschmidt J, Von Bohlen A, Liebl K, Püttmann W (2004). Concentration and distribution of platinum group elements (Pt, Pd, Rh) in airborne particulate matter in Frankfurt am Main, Germany. Environ Sci Technol.

[CR46] Zereini F, Wiseman C, Püttmann W (2007). Changes in palladium, platinum, and rhodium concentrations, and their spatial distribution in soils along a major highway in Germany from 1994 to 2004. Environ Sci Technol.

[CR47] Zereini F, Alsenz H, Wiseman CLS, Püttmann W, Reimer E, Schleyer R, Bieber E, Wallasch M (2012). Platinum group elements (Pt, Pd, Rh) in airborne particulate matter in rural versus urban areas of Germany: concentrations and spatial patterns of distribution. Sci Total Environ.

