# Supplementary material for: Anthropogenic platinum group element (Pt, Pd, Rh) concentrations in PM10 and PM2.5 from Kolkata, India
Source: Springerplus. 2016 Aug 2;5(1):1242. doi: 10.1186/s40064-016-2854-5 (PMC4970991; doi:10.1186/s40064-016-2854-5)

**SUPPLEMENTARY SECTION**

Table S1. Agilent 7700 ICP-MS operating conditions for the analysis of PM samples.

| Parameter | Value |
| --- | --- |
| Plasma mode | Normal, robust |
| RF forward power (W) | 1550 |
| Sampling depth (mm) | 8 |
| Carrier gas flow (L/min) | 0.95 |
| Dilution gas flow (L/min) | 0.15 |
| Spray chamber temperature (°C) | 2 |
| Extraction lens 1 (V) | 0 |
| Kinetic energy discrimination (V) | 4 |
| He cell gas flow (mL/min) | 4 |

Figure S1. Map showing sampling locations in and around the megacity of Kolkata, India. The locations are divided broadly into two categories: traffic junctions (black circles) and industrial estates (blue circles). The traffic junctions are selected at the heart of the city with no major industry within 10 Km radius. The industrial sites are selected in the suburbs of the city where road traffic is much less compared to the city. The map is modified from Das et al., 2015.


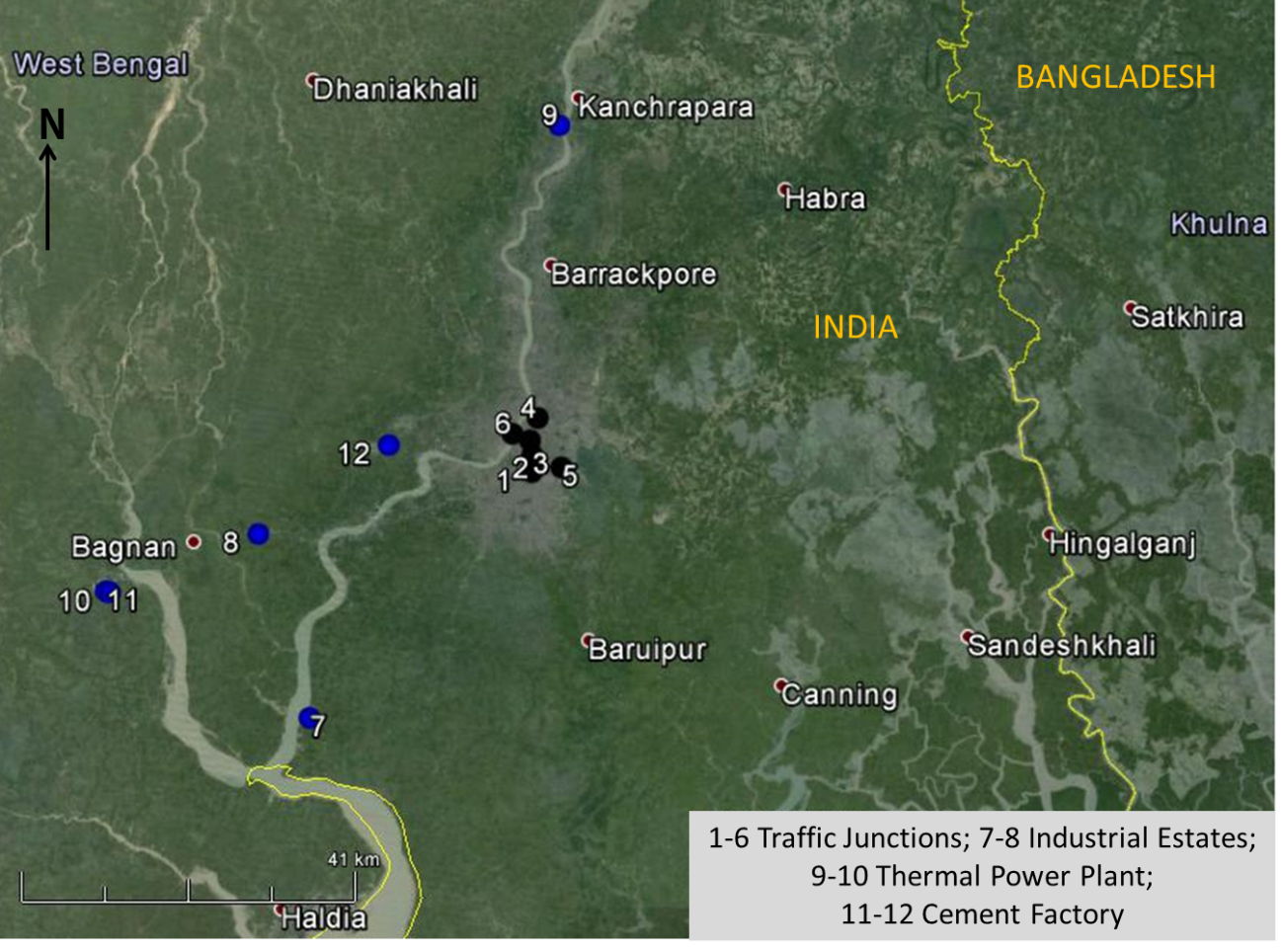

Supplement: Supplementary file 1 — 10.1186/s40064-016-2475-z Agilent 7700 ICP-MS operating conditions for the analysis of PM samples. Figure S1. Map showing sampling locations in and around the megacity of Kolkata, India. [file 40064_2016_2854_MOESM1_ESM.docx]
